# Coping with breast cancer during medical and occupational rehabilitation: a qualitative study of strategies and contextual factors

**DOI:** 10.1186/s12905-024-03012-3

**Published:** 2024-03-19

**Authors:** Paula Heidkamp, Kati Hiltrop, Clara Breidenbach, Christoph Kowalski, Holger Pfaff, Franziska Geiser, Nicole Ernstmann

**Affiliations:** 1 University Hospital Bonn, Department for Psychosomatic Medicine and Psychotherapy, Center for Health Communication and Health Services Research, Bonn, Germany; 2 University of Cologne, Faculty of Medicine and University Hospital Cologne, Institute of Medical Sociology, Health Services Research and Rehabilitation Science, Chair of Health Services Research, Cologne, Germany; 3 University Hospital Bonn, Center for Integrated Oncology, Bonn, Germany; 4 German Cancer Society, Berlin, Germany; 5 University of Cologne, Faculty of Human Sciences & Faculty of Medicine and University Hospital Cologne, Institute of Medical Sociology, Health Services Research and Rehabilitation Science, Chair of Quality Development and Evaluation in Rehabilitation, Cologne, Germany; 6 University Hospital Bonn, Department of Psychosomatic Medicine and Psychotherapy, Bonn, Germany

**Keywords:** Cancer, Cancer survivorship, Coping, Oncology, Qualitative, Rehabilitation, Return to work

## Abstract

**Purpose:**

This study aimed to gain a deeper understanding of the coping processes of breast cancer survivors (BCSs) during medical and occupational rehabilitation after acute treatment.

**Methods:**

This study is part of the mixed-methods Breast Cancer Patients’ Return to Work study conducted in Germany. Data were collected through semistructured interviews with 26 female BCSs 5–6 years after their diagnosis. A qualitative content analysis was conducted to investigate the coping strategies and contextual factors of coping of BCSs.

**Results:**

The participants used different strategies for coping with their breast cancer, namely, *approach- versus avoidance-oriented coping* and *emotion- versus problem-focused coping*. During the medical rehabilitation process, coping behavior was used mainly to address disease management and its consequences. During the occupational rehabilitation process, most coping strategies were used to overcome discrepancies between the patient’s current work capacity and the job requirements. The contextual factors of coping were in the health, healthcare, work-related, and personal domains.

**Conclusion:**

The study findings provide in-depth insights into the coping processes for BCSs during the rehabilitation phase and highlight the importance of survivorship care after acute cancer treatment.

**Implications for Cancer survivors:**

The results indicate that BCSs employ approach- and avoidance-oriented strategies to cope with their cancer during rehabilitation. As both attempts are helpful in the short term to cope with physical and emotional consequences of the cancer, healthcare and psychosocial personnel should respect the coping strategies of BCSs while also being aware of the potential long-term negative impact of avoidance-oriented coping on the rehabilitation process.

## Introduction

Breast cancer (BC) is the most common cancer among women in Germany, with almost 70,500 newly diagnosed cases annually [1]. Screening programs and treatment advances have increased these patients’ chance of early diagnosis and survival rate [1]. About 30% of these patients are 59 years old or younger [1] and thus in the working-age group. Thus, it is imperative to not only restore physical and mental abilities but also reinstate the ability to work for BC survivors (BCSs) after acute treatment. The rehabilitation phase after acute cancer treatment is characterized by the reintegration into social roles while presenting various challenges for patients, such as feeling alone with treatment-related symptoms, struggling with a different self-perception and changes in personal relationships, and returning to work, along with associated worries, such as concerns regarding one’s performance limits [2, 3]. Furthermore, after completing acute treatment, cancer survivors (CSs) still report lower quality of life than the general population [4, 5] and considerable psychological distress [6]. To cope with their illness after acute treatment, BCSs employ different strategies [7, 8].

According to the transactional model of stress, *coping* is defined as “ongoing cognitive and behavioral efforts to manage specific external and/or internal demands that are appraised as taxing or exceeding the resources of the person” (R. S. Lazarus, [9], p. 237). Roesch et al. [10] suggested a literature-based taxonomy to classify the coping strategies of patients with prostate cancer around two dimensions: *approach- versus avoidance-oriented coping* and *emotion- versus problem-focused coping*. *Approach-oriented coping* refers to coping activity oriented toward a threat, such as seeking information, whereas *avoidance coping* refers to an attempt to direct attention away from a threat, such as by denial [10]. *Emotion-focused coping* aims to regulate the emotional consequences of a stressful situation, such as by positive reinterpretation [11], whereas *problem-focused coping* is the active attempt to influence the source of stress, such as by seeking instrumental support.

Coping style is relevant among BCSs as different patterns predict psychological symptoms and quality of life outcomes, even years after the diagnosis [12, 13]. Compared with approach-oriented coping, avoidance-oriented coping exerts an adverse effect and is associated with lower quality of life and worse physical and psychological health [10, 12, 14–16]. To support patients with cancer who employ coping strategies with a potential negative impact on long-term quality of life, an understanding of contextual factors that influence coping style is critical. Quantitative studies on the predictors of coping in cancer patients and survivors found significant effects of education, age, sex, therapy, social support, and marital status [17–19]. However, specific knowledge of coping strategies and contextual factors is scarce for BCSs during rehabilitation. There is some evidence that patients with cancer who participate in an inpatient oncological rehabilitation program are more active in managing their illness than nonparticipants and that rehabilitation exerts positive effects on emotional stabilization, anxiety reduction, and resource strengthening for cancer patients [20]. Therefore, participation in a rehabilitation measure is assumed to exert a positive effect on how patients deal with their illness. However, to date, coping among CSs has not been a focal point of qualitative research [21]. Thus, this study aimed to gain a deeper understanding of coping processes among BCSs in Germany after acute cancer treatment during medical and occupational rehabilitation by analyzing coping strategies and contextual factors using qualitative interview data from BCSs 5–6 years after diagnosis.

## Materials and methods

### Study design

This study is part of the mixed-methods BC Patients’ Return to Work (B-CARE) study conducted in Germany [22]. Interview and survey data were collected 5–6 years after diagnosis to explore the rehabilitation of BCSs; however, this study focused solely on the interview data, particularly on medical and occupational rehabilitation. The definition of these phases is based on the interviewees’ subjective understanding of medical and occupational rehabilitation. Regarding *medical rehabilitation*, the experiences reported by patients relate to the period after acute treatment, mainly associated with the completion of chemotherapy and radiotherapy at the cancer center. During this period, interviewees either participated in an oncological rehabilitation measure or did not participate and instead pursued other activities to restore health. The *occupational rehabilitation* phase involves the process of resuming work after the diagnosis. The University of Bonn Ethics Committee of the Medical Faculty approved this study (approval number: 316/18; German Clinical Trials Registry number: DRKS00016982).

### Recruitment and sampling

The B-CARE study is a follow-up to the PIAT study (Strengthening Patient Competence: Breast Cancer Patients’ Information and Training Needs) and represents a subsequent survey of the PIAT sample. The preceding PIAT study aimed to explore the information needs of BC patients. A total of 1359 patients initially diagnosed with BC were recruited from 60 BC centers throughout Germany [23] and were surveyed at three measurement time points: during hospitalization (T1), 10 weeks after hospital discharge (T2), and 40 weeks after hospital discharge (T3). The follow-up B-CARE study aimed to investigate the long-term rehabilitation process of BCSs. To this end, the existing longitudinal PIAT data was utilized, and an additional measurement time point for a survey and qualitative interviews, 5–6 years after diagnosis (T4), was added. The PIAT participants who consented to be recontacted and were working at the time of diagnosis were invited to participate in the follow-up B‐CARE study 5–6 years later. A total of 184 BCSs participated in the B-CARE survey. Those who had provided written consent for an additional interview were invited via telephone or email and were informed about the procedure (audio recording, data use) and subsequently provided informed consent. Regarding the selection of interviewees, purposive sampling was employed [24]. The sampling strategy aimed to include contrasting cases with characteristics considered to be relevant to the research focus. Quantitative survey data were utilized to select interviewees with differences in sociodemographic characteristics (e.g., age, family status), rehabilitation experiences (e.g., participation/nonparticipation in an inpatient oncological rehabilitation program after acute treatment), and occupational variations (e.g., return to work after treatment, job changes that occurred). The sampling process continued until data saturation was reached [25].

### Data collection

Data were collected through semistructured interviews via telephone or in person between August 2019 and August 2020 in the participant’s preferred location, mainly at home. The interviews were audiotaped and lasted 53 min on average. The interview guide included 12 guiding open-ended questions and discussion of medical and occupational rehabilitation topics, coping strategies, and fear of cancer recurrence. In addition to the guiding open-ended questions, the interview guide included follow-up questions that could be asked if necessary. Examples of leading open-ended and follow-up questions are as follows: (1) Leading open-ended question: “Why don’t you tell us how it came about that you did not take advantage of rehabilitation measures?” Follow-up question: “What concerns did you have?” (2) Leading open-ended question: “What helped you cope with your illness?” Follow-up question: “Did you seek help? In what form?” To improve the understandability and suitability of the interview guide, two cognitive pretests were conducted. The interviews were conducted by two research assistants (KH, PH).

### Data analysis

The interview materials were transcribed verbatim. For data management, transcripts were entered into the software program ATLAS.ti (ATLAS.ti Scientific Software Development GmbH, Berlin, Germany) and analyzed after a qualitative content analysis according to the method described by Kuckartz [26]. All 26 transcripts were read, and the relevant text passages were marked. A deductive coding scheme was established according to the method described by Roesch et al. [10] with two coping categories: approach-/avoidance-oriented and emotion-/problem-focused. The transcribed interviews were reviewed from beginning to end, and relevant sections of the text were assigned to the main categories. The units of meaning were coded, which could also comprise several sentences or paragraphs. The text sections with the same main categories were compiled. Then, the main categories were differentiated by assigning them subcategories. Subcategories (coping strategies) were coded deductively inspired by the work of Roesch et al. [10, 27], the COPE [28], the Coping Responses Inventory [29], and the Ways of Coping Questionnaire [11] and were complemented by inductively derived codes. All materials were coded using the resulting coding system. If necessary, text passages could also be assigned to multiple coping strategies. Furthermore, the data were coded regarding secondary information that were relevant in the context of coping behavior (e.g., health- and work-related characteristics). The main categories (e.g., health-related contextual factors) and subcategories (e.g., participation in a rehabilitation program) were inductively coded. Subsequently, the contextual factors of coping strategies were analyzed by investigating the associations between the subcategories that emerged (coping strategies) and secondary information. To ensure reliability, the data were coded by two scientists (PH, KH). Any coding differences were discussed until consent was reached. Typical quotes were selected to illustrate the results. Filling words and duplications were omitted to increase readability.

## Results

### Sample

A total of 26 interviewees were selected using purposeful sampling. Their average age was 57 years, and most of them were married and had a part-time employment during the time of the interview. They were first diagnosed with BC in 2013, primarily stage 1 or 2. Table 1 presents the sample characteristics at the time of the interview.

**Table 1:** Sample characteristics of the 26 interviewees

Characteristics		Interviewees (*n* = 26)	Mean	Min–max
Age in years	Missing	0	56.73	44–72
Marital status	Married	18		
	Single	4		
	Divorced	3		
	Widowed	1		
	Missing	0		
Children	Yes	17		
	No	7		
	Missing	2		
Vocational training	No training	1		
	Vocational training	9		
	Specialized/master Craftsman training	4		
	University	11		
	Missing	1		
Employment status	Full-time	8		
	Part-time	13		
	Retired†	5		
	Missing	0		
Rehabilitation program participation after acute treatment of initial breast cancer	Yes	19		
	No	7		
UICC TNM stage of initial breast cancer ‡	0	2		
	1	11		
	2	8		
	3	1		
	Missing	4		
Recurrence	No	21		
	Yes	5		
	Missing	0		

† Includes early retirement and reduced earning capacity retirement.

‡UICC TNM = Union for International Cancer Control TNM staging [30]

### Coding trees

**Fig. 1:**
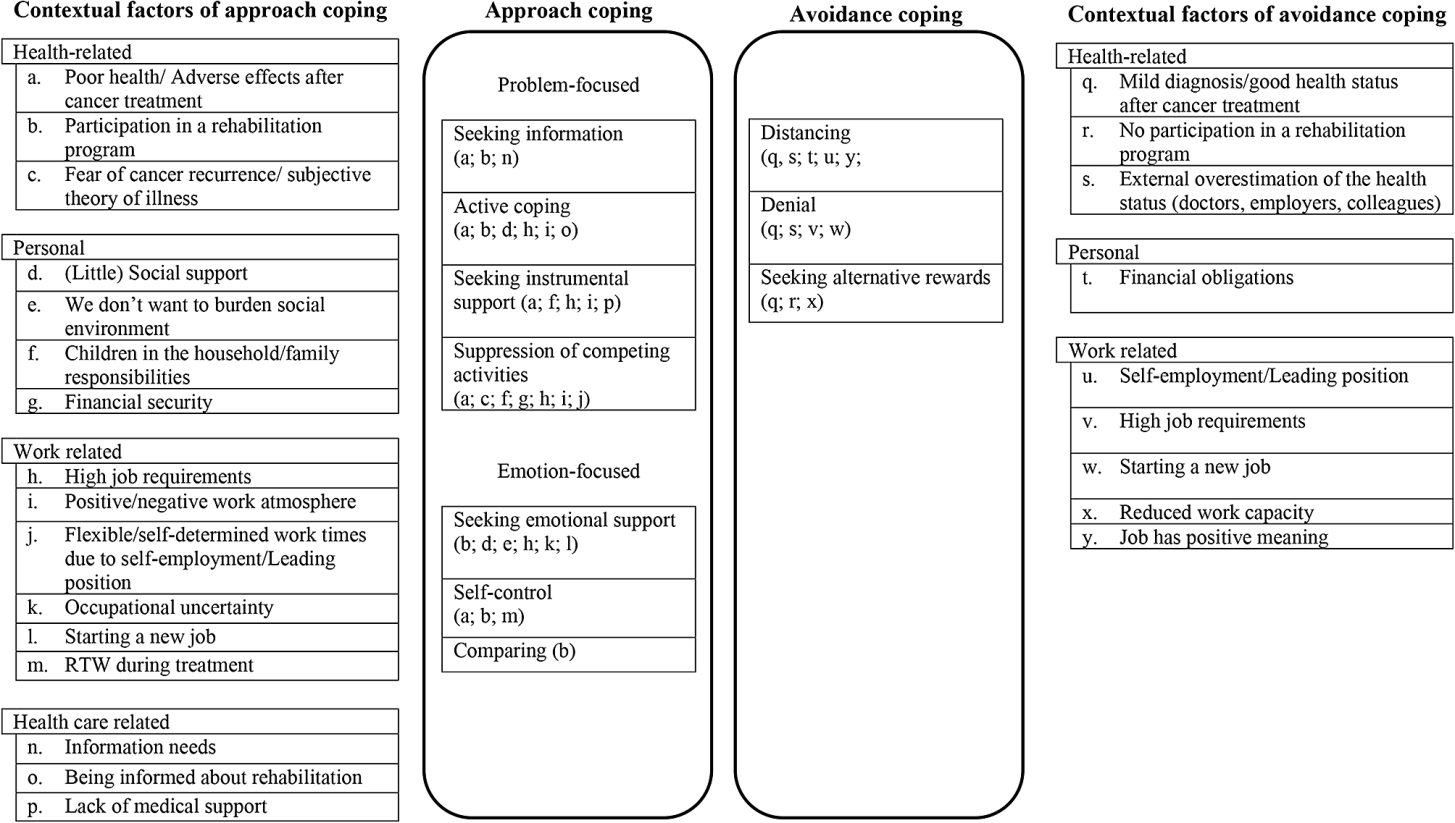
Coping strategies used by breast cancer survivors and associated contextual factors during medical and occupational rehabilitation. *Note.* The associated contextual factors are shown in brackets after the coping strategies

During medical and occupational rehabilitation, BCSs employed different coping strategies, classified as either approach- or avoidance-oriented coping. Approach-oriented coping involved problem-focused coping strategies of *seeking information*, *active coping*, *seeking instrumental support*, and *suppression of competing activities* and emotion-focused strategies of *self-control*, *seeking emotional support*, and *comparing*. In avoidance-oriented coping, BCSs employed the strategies of *distancing*, *denial*, and *seeking alternative rewards.* We also analyzed the contextual factors of coping strategies to gain a better understanding of coping behavior and associated factors. The theoretical foundation for the investigation of contextual factors is based on the work of Mehnert et al. [31] in which contextual factors associated with return to work of CSs were studied. We identified contextual factors in the health, healthcare, work-related, and personal domains. Figure 1 presents the approach- and avoidance-oriented strategies and associated contextual factors.

### Approach-oriented coping

#### Problem-focused coping

***Seeking information*** was identified as a problem-focused strategy employed by BCSs to cope with their illness during rehabilitation. The use of this strategy is motivated by existing information needs and promoted by participating in an oncological rehabilitation program. BCSs sought information regarding long-term adverse effects, future perspectives toward the cancer, and means to have a positive impact on the prognosis. Interviewees found it helpful to receive information from other BCSs who had more time since their diagnosis or had a cancer relapse, for example, participant 3 (P3) in an inpatient rehabilitation program:

*“There were also a lot of people there who had fallen sick again. And that’s something EVERYONE is afraid of, right? And then you got to hear, how it was developing now, what are their chances? You didn’t know how the disease was progressing, right? I thought it was good.” —P3*

Seeking information helped normalize the interviewees’ experiences, gain a clearer picture of the future disease course, cope with worries, and create a sense of control and self-efficacy.

***Active coping*** is an attempt for active rehabilitation posttreatment, including exercise, healthy eating, participation in therapies (e.g., lymph drainage, physiotherapy), informative meetings, psychological counseling, and willingness to “do whatever it takes” to rehabilitate. It often manifests as participation in organized inpatient rehabilitation programs and was reported by interviewees who were informed about the possibility of active rehabilitation (e.g., in a rehabilitation program), who were motivated by a supportive social environment, and who experienced poor health posttreatment. Women who had physical impairments considered it more necessary to actively engage in rehabilitation than those with subjective good health status, as explained by P12 speaking about her motivation to participate in a rehabilitation program:

*“actually the physical condition. Rather than the mental or psychological state because I had so many side effects from the chemo, I was not mobile at all and always felt tired. That was really the aspiration* [sic].*” —P12*

Active coping is also employed for occupational rehabilitation to cope with discrepancies between job requirements and an impaired capacity to work because of adverse treatment effects. It involves making adjustments actively, such as incorporating recovery time in the work routine or openly communicating about the illness to workers and employers. Interviewees with high job requirements as well as social support and understanding from colleagues/employers reported using active coping. This strategy also includes seeking a new job when the current position becomes incompatible with rehabilitation and was reported in connection with performance pressure as well as the lack of support and understanding at the workplace.

***Seeking instrumental support*** is defined as seeking of support from family and friends who care for the household or interviewees’ children to enable active rehabilitation. Many interviewees sought instrumental support for continuing health problems and “inexplicable” treatment-related symptoms. They consulted rehabilitation clinic physicians and other healthcare providers (e.g., osteopaths, acupuncturists) as well as self-help groups for instrumental support. Interviewees who perceived a lack of medical support and felt devalued and neglected by physicians during treatment used this coping strategy, as reported by P6 about healthcare deficits that motivated her to seek instrumental support in a rehabilitation clinic:

*“I already noticed that I was in pain, that it was indefinable and all a mystery and my doctors actually always told me that it doesn’t exist, that the pain will go AWAY again. So they were actually negating everything or talk me into it. And I was really hoping to find someone there in the treatment center who would help me in a really HONEST way.” —*P6

Seeking instrumental support also affected occupational rehabilitation and helped overcome discrepancies between job demands and impaired work capacity. Therefore, during return to work, interviewees sought support from colleagues/employers who undertook certain tasks to relieve them. Another manifestation of instrumental support is progressive reintegration, which enables employees to gradually increase their working hours after sick leave. Interviewees reported seeking instrumental support in connection with poor health posttreatment, high job requirements, as well as understanding and support from supervisors and colleagues.

***Suppression of competing activities*** is defined as suppression of activities competing with self-care to focus on rehabilitation and recovery time. During medical rehabilitation, this strategy especially manifests as suppression of family duties and was reported by interviewees with children in the household and family responsibilities, as described by P19, an inpatient rehabilitation participant:

*“I was really focused on myself there. I did [n’t] miss my family either. That was good, I can say now that I was happy to get rid of them (laughs). And because I also had the freedom to think about things myself.” —* P19

Suppression of competing activities also occurs in the context of occupational rehabilitation, during which work obligations are adapted for impaired work capacity and health conditions posttreatment. This work subordination manifests as reduced work time, changes in work scope, stronger focus on work/life balance, and job changes. Suppression of work in favor of health activities was reported in connection with the long-term adverse effects of treatment (e.g., fatigue, joint pain), high job requirements, and support and understanding in the workplace, as reported by P19 about making adjustments due to reduced work capacity posttreatment:

*“I’m also really grateful to my boss at the time, we agreed that I’d be working successfully again after my reintegration, but basically for a period of two years I’d be doing a job in which I was no longer exposed to maximum stress. And I wouldn’t have been able to do the job anyway anymore because with a job like that, you don’t know when you go to work in the morning, how long the day will be and what the day will bring. That means I wouldn’t have been able to do the job under those conditions any more—i.e., with the background and in the physical condition I was in when returning to work.” —* P19.

Furthermore, financial security, particularly with married status, and having flexible/self-determined work times due to self-employment or leading positions were associated with need-oriented adaptation of work obligations in favor of health aspects. Other factors in this coping strategy are fear of cancer recurrence and a subjective theory of illness in which work stress is perceived as the cause of the cancer.

### Emotion-focused coping

***Seeking emotional support***—particularly from fellow patients in the context of rehabilitation programs or self-help groups—is a strategy employed by many of the interviewees. Being with patients who had similar experiences made the interviewees feel understood, normalized their own feelings and perceptions, and provided them with an opportunity to express their feelings. Interviewees reported seeking emotional support from fellow patients as they did not want to burden their personal social environment or they had little social support at home. During medical rehabilitation, the interviewees sought emotional support from professionals (e.g., psychooncologists, psychotherapists, and physicians) to cope with the emotional impact of their diagnosis and its consequences. Emotional support is also sought for the psychological burden of occupational rehabilitation, particularly for emotional distress when work return is impossible due to impaired capacity or another reason, specifically to cope with uncertainty regarding the occupational future and challenges of a new job. This context especially includes feeling unable to transparently communicate about the cancer at the new workplace and dissimulation, leading to external expectations for high performance and resulting in overexertion and work overload. This association is described by P8 who started a new job posttreatment and sought emotional support:

*“after I went back to WORK, I felt like I was having like a panic attack. I couldn’t really explain it. It was also like that while I was working. And nobody at the new job knew what was wrong with me. There were two or three situations, I can remember, where I had to struggle with myself. And then I discussed it with my gynecologist. And we thought about how to deal with it. Whether it might make sense to seek psychotherapeutic support in some way.” —* P8

***Self-control*** is an attempt to regulate one’s emotion, be strong, and not let negative feelings affect one’s behavior. This strategy positively affects rehabilitation because it helps overcome reluctance, such as participation in oncological rehabilitation while wishing to stay at home with the family. Self-control is used during occupational rehabilitation to support return to work and regain normalcy despite not feeling emotionally or physically prepared. Interviewees who used self-control were those who reported more serious health issues posttreatment and those who returned to work during treatment.

***Comparing*** is a strategy based on downward comparisons with fellow patients, particularly in the context of inpatient rehabilitation. This strategy helped the interviewees accept their health condition and led to feelings of thankfulness and luck compared with others, as described by P12:

*“There were, of course, other patients there who were going through something similar. And then you were able to see that things could always be WORSE, right? That’s always a consolation or motivation somehow.” —* P12

### Avoidance-oriented coping

***Distancing*** is an attempt to draw attention away from being ill, to remove oneself from the “sick” role, and to separate from emotions related to cancer. This strategy is motivated by a desire for normalcy and wish to move beyond cancer.

Regarding medical rehabilitation, distancing includes avoidance of rehabilitation programs and of fellow patients. Distancing was reported in connection with a milder diagnosis by interviewees concerned about being burdened rather than supported by fellow patients. Thus, subjective good health facilitated this strategy.

Distancing also plays a pivotal role in occupational rehabilitation. Returning to work helps draw attention away from cancer and creates normalcy, especially if the job is positively connoted as a source of joy and self-worth. Distancing manifests as a work return during treatment, work return without progressive reintegration, and workplace avoidance of the issue of illness. Distancing is promoted if the BCS is externally perceived as recovered or healthy (e.g., new job colleagues are unaware of the cancer, workplace members do not discuss illness). This association is illustrated by P2 who returned to work during treatment and spoke about her colleagues’ support:

*“And because I then took on some OTHER tasks, the two colleagues I joined in the office turned out to be two young men, and men take things differently to women anyway, right? They don’t talk about it [the illness] much at all, which made it EASIER for me because I didn’t come to work to explain all sorts of details about chemotherapy; rather, when you’re there, you’re there and men deal with this more objectively. And they really made the beginning easy for me.” —* P2

Distancing from the sick role at the workplace was reported to be associated with self-employment, having a leading position, and having financial obligations (e.g., paying off debt).

***Denial*** refers to disclaiming physical impairments and symptoms and overestimating one’s fitness and work ability to regain normalcy and move beyond cancer. Thus, it leads to refusal of organized medical or occupational rehabilitation programs and return to work with the same prediagnosis workload, resulting in physical and occupational overload. In retrospect, interviewees were able to reflect on denial, as noted by P3:

*“So looking back, I think I wasn’t yet 100% ready for work. I pretended I was, right? I have quite got my head around it, right? There were still things that needed to be done somehow. So it’s hard to explain now, in retrospect. If you’d have asked me back then: ‘Yes, I’m back again in top form’, right?” —* P3

This strategy was reported in connection with a milder diagnosis, external overestimation of health status, high job requirements, and starting a new job posttreatment, leading to perceived incompatibility with the sick role. External assessment by physicians or family may promote denial, as noted by P17:

*“Of course, I’d also ask the doctor if it was okay [to go to the football match]. And then they said “Yes, if you feel OK, why shouldn’t you go, right?” Yes. And then I went with the others. … and that then set everything off, of course. It was all too much, of course. But I didn’t see it like that at all myself. So I didn’t realize at all at myself, how sick I actually was. And how weak I actually am. …I didn’t even notice that I was doing so much above and beyond the strength I had. And that was the reason why I didn’t do any rehab either. Because I thought No, you’re not that sick. Then, at the hospital, a doctor said “Yes, sometimes it’s not good either, because there are a lot of people there who really are in a poor shape. And then you let yourself get dragged down even more, psychologically.“… And I didn’t realize that at all, that something actually could have been done.” —* P17

***Seeking alternative rewards*** is an attempt to direct one’s attention away from the cancer and toward a source of positive feelings, such as joy and appreciation. Alternative rewards include vacation and positive activities such as enjoying culture and nature. This strategy helps recovery from disease and treatment. Interviewees who reported using this approach were those who refused inpatient rehabilitation and who had a milder diagnosis, resulting in subjective good health posttreatment. Seeking alternative rewards also comprises engagement in voluntary work, associated with reduced work capacity posttreatment. Voluntary work provides an opportunity to “give back” within the BCS’s capacity and to make them feel useful and appreciated.

## Discussion

This study investigated the coping processes of BCSs during rehabilitation and analyzed contextual factors. It was found that the interviewees used different coping strategies, classified as approach- or avoidance-oriented coping. The classification of coping strategies was based on the taxonomy by Roesch et al. developed for patients with prostate cancer. To the best of our knowledge, we only found one taxonomy in literature for categorizing coping strategies, specifically for patients with BC and BCSs [15]. Kvillemo et al. suggested a taxonomy that categorizes coping strategies at a higher level into engagement coping, comparable to approach coping, disengagement coping, comparable to avoidance coping, and miscellaneous coping strategies. Engagement coping is further divided into primary control coping, which includes strategies to change the stressor or related emotions, and secondary control coping, which pertains to strategies that facilitate adaptation to stress. Both taxonomies are very similar at a higher level; however, Roesch’s model was preferred over Kvillemo et al.’s model for the categorization of coping strategies owing to its simplicity.

During medical rehabilitation, coping behavior mainly targets cancer management and its physical and emotional consequences, whereas coping strategies in occupational rehabilitation focus on overcoming discrepancies between job requirements and current work capacity, either by problem-focused coping with suppression of competing activities or avoidance such as denial.

The challenge for BCSs in balancing their disease and job demands posttreatment has also been described by Hiltrop et al. [32]. These authors found that BCSs perceive conflicts between cancer management and other life demands, including work. To cope with conflicting demands, BCSs tend to make sacrifices to the detriment of work [32]. These findings are consistent with our results regarding the coping strategy of suppression of competing activities.

Coping strategies encompass both dispositional and situational aspects, and dispositional tendencies can influence situational coping behavior [33, 34]. Thus, both aspects likely play a role in the coping processes investigated in this study. As we sought to understand how BCSs cope with consequences of the cancer during a specific phase of the cancer journey, we focused more on the situational aspects of coping. It is likely that coping strategies vary across the different phases of the cancer journey, each presenting unique challenges [35–37]. The results provide knowledge about a specific coping during the rehabilitation phase, which is characterized by the challenge for BCSs in processing the preceding phases (diagnosis, acute treatment) while simultaneously reintegrating into social roles and normalcy.

We also analyzed the contextual factors of coping in the health, healthcare, work-related, and personal domains. Regarding health-related factors, our results indicate that poor health and long-term adverse effects (e.g., fatigue) posttreatment promote approach-oriented coping. Contrarily, avoidance-oriented coping is associated with a milder diagnosis, resulting in subjective good health posttreatment. The results indicate that during rehabilitation, physical and mental impairments necessitate active and problem-focused coping; conversely, the absence of major health issues enables BCSs to distance from the sick role and promote avoidance-oriented coping. As avoidance can reduce chances for adequate rehabilitation, a long-term negative impact on the quality of life or work may be expected, as reported in previous studies in which avoidance- versus approach-oriented coping in cancer patients was associated with lower quality of life as well as worse physical and psychological health [10, 12, 14]. However, it should also be noted that in some cases, the decision to not participate in rehab or engage in other forms of active coping may be based on a realistic assessment of one’s own state of health and performance and does not always represent an avoidance-oriented coping strategy.

In addition to the interviewees’ self-perception regarding their health status posttreatment, external perceptions of others played a role in coping behavior. Avoidance-oriented coping was associated with relativizing medical opinion and being perceived as recovered or healthy by colleagues/employers. Thus, external assessment overestimating the health of BCSs may promote avoidance-oriented coping (e.g., an employer offering promotion during treatment) and may be the result of avoidance-oriented coping (e.g., a BCS’s self-distancing from cancer).

Our findings indicate an association between coping style and participation in an oncological rehabilitation program. Interviewees who employed approach-oriented coping strategies were more likely to participate in a rehabilitation program. In addition, the context of a rehabilitation program enabled the use of certain approach-oriented coping strategies, such as comparing. Therefore, participating in oncological rehabilitation may represent active coping with physical and emotional consequences of the cancer and be a contextual factor that facilitates approach-oriented coping. Notably, BCSs in Germany who wish to apply for early retirement due to cancer must first undergo rehabilitation. Furthermore, in this case, participation in rehabilitation represents an active coping behavior to deal with the illness. Simultaneously, avoidance-oriented coping seems to be a barrier to rehabilitation program participation. This finding is consistent with the results of other studies [38, 39]. Deck et al. [40] analyzed the reasons for the nonuse of oncological rehabilitation of CSs, the most frequent being desire for normalcy, distance from the cancer, and avoiding fellow patients.

Healthcare deficits, such as existing information needs and perceived lack of medical support, were associated with approach-oriented coping strategies such as seeking information and instrumental support. Our findings indicate that approach-oriented coping may mitigate the impact of healthcare deficits, which is supported by Ahadzadeh and Sharif [41] who observed a moderating effect of approach-oriented coping on the negative association between information needs and quality of life in patients with BC.

Regarding work-related contextual factors, our findings suggest that support and understanding in the workplace promote problem-focused coping (e.g., seeking support from colleagues) to overcome discrepancies between job demands and work capacity. Thus, a supportive work atmosphere may be a facilitating factor in work return and contribute to successful occupational reintegration. Jin and Lee [42] supported this assumption as they found a positive effect of workplace social support on the quality of work life among CSs who returned to work. Hiltrop et al. [43] reported a positive association between the *social capital of the workplace*, which describes workplace-related aspects such as trust or common values [44], and BCSs’ satisfaction with their occupational development 5–6 years after the diagnosis.

Other contextual factors associated with coping behavior are self-employment or a leading position in the workplace. Both facilitates suppression of competing activities to adapt work life to health-related needs, such as making time for rehabilitation sports during the work day or reducing work hours. This association may be explained by the possibility of scheduling working times more flexibly and autonomously. Simultaneously, avoidance-oriented coping such as distancing was reported in connection with self-employment or having a leading position. These findings are consistent with those of other studies that demonstrated an association between self-employment and the opportunity to flexibly work with an earlier return to work for CSs [45, 46]. In addition to flexible working hours, this association may be explained by financial necessity and a perceived responsibility for clients and employees, making it more necessary to distance from the sick role.

Regarding personal factors, our findings suggest an association between approach-oriented coping and social support. The presence of a supportive social environment may promote an active coping style (e.g., by motivating the BCS to participate in rehabilitation). At the same time, approach-oriented coping is employed to cope with a perceived lack of social support, such as seeking emotional support from fellow patients. Another contextual factor is financial status. Financial security, often associated with being married, allowed interviewees to suppress work activities in favor of health aspects, such as by reducing work time. Contrarily, financial obligations (e.g., debt) promoted avoidance-oriented coping strategies, such as distancing. Financial security may thus be a facilitating factor for rehabilitation, whereas financial obligations may be a barrier to the rehabilitation process.

### Study limitations

Our study results provide a better understanding of the challenges, coping behaviors, and contextual factors of rehabilitation after BC. Several study limitations must be considered when interpreting these results. Because of the qualitative approach, the generalizability of the results is limited. This especially applies to the associations observed between the contextual factors and coping behavior of BCS. The study samples consisted of female BCSs who were employed before diagnosis and did not include male CSs or other tumors. Because all interviews were conducted in Germany and in the context of the specific German system of rehabilitation, the experiences of BCSs may differ from those in other healthcare systems. The interviews were conducted 5–6 years after diagnosis; thus, effects of recall bias are possible. However, the rehabilitation phase after cancer may be a salient life experience that reduces the memory effects.

### Clinical implications

This study provides in-depth insights into the coping process of BCSs during rehabilitation. The results indicate that BCSs employ approach- and avoidance-oriented strategies to cope with their cancer during rehabilitation. As both strategies are helpful in the short term to cope with the physical and emotional consequences of the cancer, healthcare and psychosocial personnel should respect BCSs’ coping strategies while also being aware of the potential long-term negative impact of avoidance-oriented coping on the rehabilitation process. Health and psychosocial personnel in inpatient and outpatient settings (e.g., cancer counseling centers) should speak openly to BCSs about their coping behavior and inform them about the possible long-term risks of avoidance-oriented coping. To support BCSs in coping with their illness more flexibly, information needs (e.g., regarding rehabilitation programs) should be reduced and fears (e.g., being burdened by fellow patients during rehabilitation) should be addressed. The findings regarding contextual factors for coping may help screen BCSs in inpatient and outpatient settings for disadvantageous circumstances (e.g., financial obligations, starting a new job posttreatment) and to support those engaged in a rehabilitation process. Furthermore, increasing employers’ awareness of the challenges of returning to work after cancer may positively impact the occupational rehabilitation of BCSs. The literature shows that there is a lack of interventions aimed at sensitizing employers and coworkers to the needs of CSs and improving communication, thereby supporting the professional reintegration of CSs [47, 48].

## Data Availability

The datasets generated and/or analyzed during the current study are not publicly available due to the patient consent form but are available from the corresponding author on reasonable request.
